# Chemical Contaminants in Drinking Water: Where Do We Go from Here?

**DOI:** 10.1289/ehp.122-A80

**Published:** 2014-03-01

**Authors:** Julia R. Barrett

**Affiliations:** Julia R. Barrett, MS, ELS, a Madison, WI–based science writer and editor, has written for *EHP* since 1996. She is a member of the National Association of Science Writers and the Board of Editors in the Life Sciences.

Given the number of chemicals in the environment and people’s variability in exposure and susceptibility to harm, it’s a daunting challenge to catalog all possible drinking water contaminants and assess their associated health risks. But after reviewing the state of the science and the data gaps surrounding drinking water contaminants, a team of authors presents in this issue of *EHP* an ambitious roadmap to help future studies identify and elucidate risks presented by specific contaminants.^1^

Although microbial agents are the largest cause of waterborne diseases worldwide,^1^ chemical contaminants in drinking water have been associated with a broad array of adverse health effects, including cancer, cardiovascular disease, neurological disease, and miscarriage.^2^ Some contaminants enter water through leaching, accidental spills, runoff, and atmospheric deposition. Others, such as disinfection by-products and lead, are introduced during treatment or even at the tap.^3^

Contaminants may occur naturally, or they may derive from human-related activities, such as industry, agriculture, and mining. Not only have the prevalence and uses of chemicals escalated in the last century, but the analytical techniques to detect them have become exquisitely sensitive.^4^^,^^5^ Consequently, it is possible to define vanishingly small levels of both well-studied and emerging contaminants in drinking water.

The presence of a contaminant does not necessarily translate to an adverse impact on human health; the levels may be unimportant, exposure is not a given, and toxicity may depend on individual susceptibility. Assessing the human health effect of any given chemical requires careful epidemiological and toxicological study, which has not been conducted for most drinking water contaminants.^1^

**Figure:**
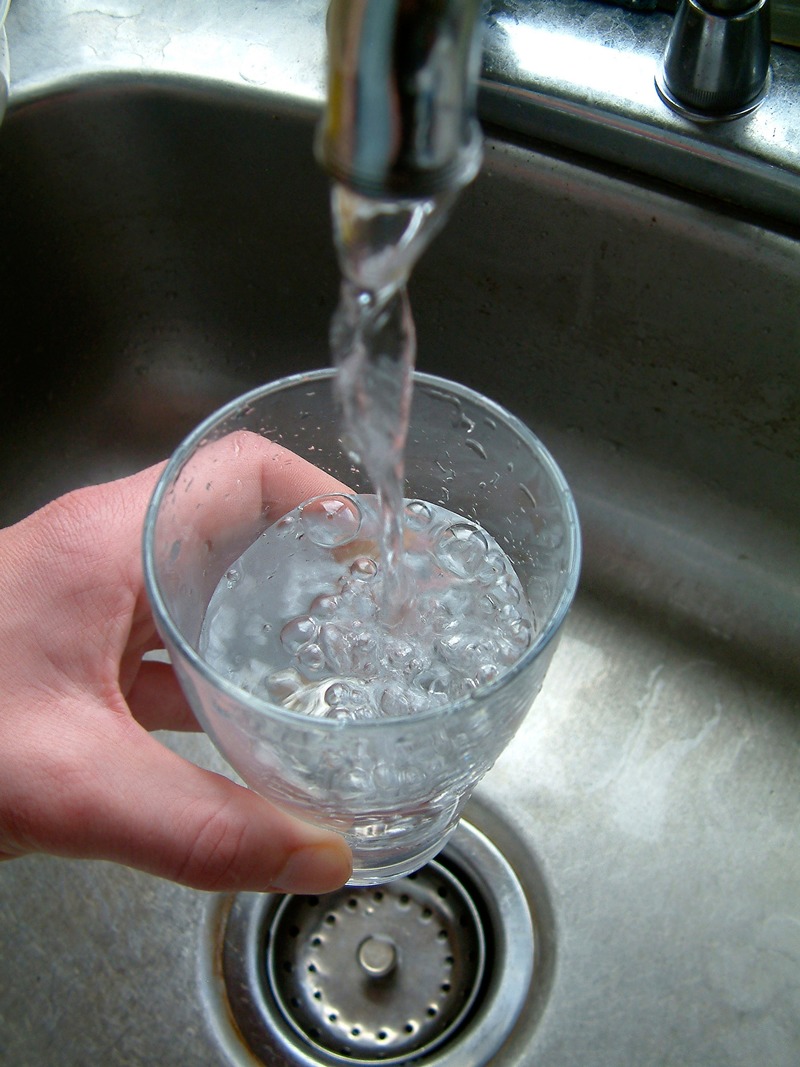
Longitudinal cohort studies can help address drinking water data gaps by incorporating water consumption in their design. © jeu/iStock

“The amount that we know about chemicals is really very little,” says Herman Gibb, president of research consultancy Tetra Tech Sciences, who was not involved with the review. “We keep hammering on the same chemicals like arsenic, cadmium, and so forth, but there are so many chemicals that we don’t know much about. And even for the chemicals for which we have relatively good information, we still don’t know enough.”

To address the knowledge gaps, the Centre for Research in Environmental Epidemiology (CREAL) in Barcelona organized a workshop in 2012 to discuss the state of the science vis-à-vis chemical contaminants in drinking water and make recommendations for future research. “Having the workshop and writing [this review] has led to some ideas for future collaborations,” says lead author Cristina Villanueva, an associate research professor at CREAL. Ultimately, the authors’ recommendations are intended to provide a template for researching any type of chemical contaminant occurring in drinking water.^1^

The U.S. Environmental Protection Agency, World Health Organization, and European Union Council together currently regulate more than 120 drinking water contaminants.^1^ But even regulated chemicals carry some uncertainty because new information requires questioning and possibly revising limits. Emerging chemical contaminants also generate concern because little to nothing is known about their potential health effects, much less the levels at which those would occur. Likewise, contaminant mixtures may pose greater threats than their individual components.

The review authors call for specific steps to address the many unknowns. In addition to determining the identity and level of individual contaminants, which will help to prioritize research topics, investigators need information on human exposures, which could be gained through epidemiological study as well as statistical modeling. Ideally, ongoing longitudinal cohort studies would incorporate drinking water consumption in their design, and particular attention would be focused on vulnerable populations, such as children and pregnant women. Information on contaminants’ mechanisms of toxicity and biomarkers of exposure could potentially link exposure with health outcomes.^1^

Villanueva and her colleagues cite climate change as a major future challenge projected to affect drinking water quality through more frequent extreme weather events and increased growth of toxin-producing cyanobacteria.^1^ Gibb, who coauthored a 2014 review of data needs related to health effects of chemicals,^6^ adds that current trends point to increased chemical contamination of the environment, particularly in developing countries.

“It’s quite a wish list of things to do,” Gibb says of the Villanueva review. “The focus here is on drinking water, but this really applies to the chemical world in general because there’s so much that we don’t know about how it affects the burden of disease.”
